# Increasing knowledge on adolescent mental health in low- and middle-income countries: The National Adolescent Mental Health Surveys

**DOI:** 10.1186/s13034-025-00920-6

**Published:** 2025-07-31

**Authors:** James G. Scott, Holly E. Erskine, Shoshanna L. Fine, Nguyen Duc Vinh, Siswanto Agus Wilopo, Caroline W. Kabiru, Robert Wm. Blum

**Affiliations:** 1 Child Health Research Centre, The University of Queensland, South Brisbane, QLD Australia; 2 Child and Youth Mental Health Service, Children’s Health Queensland, South Brisbane, QLD Australia; 3 Queensland Centre for Mental Health Research, Wacol, QLD Australia; 4 School of Public Health, The University of Queensland, Herston, QLD Australia; 5 Institute for Health Metrics and Evaluation, University of Washington, Seattle, WA USA; 6 Department of Population, Family and Reproductive Health, Johns Hopkins Bloomberg School of Public Health, Johns Hopkins University, Baltimore, MD USA; 7 Department of Mental Health, Johns Hopkins Bloomberg School of Public Health, Johns Hopkins University, Baltimore, MD USA; 8 Institute of Sociology, Vietnam Academy of Social Sciences, Hanoi, Vietnam; 9 Center for Reproductive Health, Faculty of Medicine, Public Health, and Nursing, Universitas Gadjah Mada, Yogyakarta, Indonesia; 10 Department of Biostatistics, Epidemiology, and Population Health, Faculty of Medicine, Public Health, and Nursing, Universitas Gadjah Mada, Yogyakarta, Indonesia; 11 African Population and Health Research Center, Nairobi, Kenya

**Keywords:** Psychiatric epidemiology, Mental disorders, Prevalence, Risk factors, Measurement, Stigma

## Abstract

**Background:**

There are limited prevalence data available for mental disorders in adolescents living in low- and middle-income countries (LMICs). The National Adolescent Mental Health Surveys (NAMHS) measured the prevalence of six common mental disorders, along with self-harm and suicidal behaviours, associated risk and protective factors, and service use in adolescents aged 10–17 years in Kenya, Indonesia, and Vietnam. The challenges and opportunities arising from large scale epidemiological mental health research in low resource settings are discussed.

**Measurement of mental disorders in low- and middle-income countries:**

Diagnostic criteria for mental disorders are largely informed by evidence and experiences from high-income countries. NAMHS reports a low prevalence of adolescent mental disorders in Indonesia and Vietnam, suggesting there is much that the Global North can learn from the Global South in relation to population mental health. Improving population mental health requires a public health approach which focuses on promotion of wellbeing, increased community cohesion, and prevention of exposure to risk factors in early life.

**Conclusion:**

NAMHS significantly advances knowledge of adolescent mental health in LMICs. These data provide a baseline from which future trends of mental health in these countries can be compared. This will be increasingly important as the world faces ongoing challenges, such as conflict and climate change, which will inevitably affect global mental health.

## Background

A significant proportion of the global burden of disease in adolescents is attributable to mental disorders [1]. Almost half of all individuals with mental disorders will experience first onset of illness by 18 years of age [2]. However, few studies have examined the prevalence of mental disorders in adolescents living in low- and middle-income countries (LMICs) [3] where more than 90% of the world’s adolescent population live [4].

Utilising the Diagnostic Interview Schedule for Children, Version 5 (DISC-5), the National Adolescent Mental Health Surveys (NAMHS) measured the prevalence of six common mental disorders (social phobia, generalised anxiety disorder, major depressive disorder, posttraumatic stress disorder, attention-deficit/hyperactivity disorder (ADHD), and conduct disorder), along with 12-month and lifetime self-harm and suicidal behaviours, associated risk and protective factors, and service use in adolescents aged 10–17 years in Kenya, Indonesia, and Vietnam. All adolescent participants were provided with short definitions of suicide and self-harm before answering the relevant questions. Self-harm was defined as “deliberately hurting or injuring yourself without trying to end your life” whilst suicide or attempted suicide was “taking some action to try and end your own life”.

Adolescents and their primary caregivers were interviewed from households selected randomly according to sampling frames specifically designed to elicit nationally representative results with very high participation rates in all countries. There were striking differences in the prevalence between countries with mental disorders, self-harm, and suicidal behaviour being much less common in Indonesia and Vietnam compared to Kenya [5]. Key findings presented in this Supplement include:

1. Substantial between-country variation in the prevalence of adverse childhood experiences (ACEs) [6] and bullying victimisation and perpetration [7]. Exposure to ACEs or bullying was associated with increased risk of mental disorders.
2. A significant proportion of adolescents identified as sexually or gender diverse in Indonesia and Vietnam [8]. They had increased odds of depressive and anxiety symptoms, as well as suicidal ideation.
3. A minority of adolescents with mental disorders accessed services for emotional or behavioural problems [9]. Adolescents who were supported by caregivers or peers had significantly lower prevalence of any mental disorder in the past 12 months compared to those who perceived they had limited support [10].

The hitherto absence of national prevalence surveys in LMICs reflects the significant challenges in conducting this type of research. Multiple factors impede large epidemiological studies in LMICs, including a lack of research capacity and infrastructure, scarcity of funding and resources, within-country linguistic and cultural diversity, and the increased likelihood of political instability and conflict. NAMHS was possible through an enduring research collaboration across five countries, enabled by philanthropic financial support [11].

## Priorities

Historically, research into physical illnesses has been prioritised over mental health in low-resource settings. Consequently, disease burden estimates of mental disorders for LMICs, which inform government policies, are often modelled using approximations due to the absence of available data, leading to wide uncertainty intervals and a subsequent inability to track changes over time. Without epidemiological evidence, the burden of mental disorders in LMICs will be overlooked and the omission of mental health as a health priority perpetuated.

## Challenges of measurement

Measuring mental disorders with instruments such as the DISC-5, which assesses mental disorder diagnoses in accordance with the American Psychiatric Association’s Diagnostic and Statistical Manual of Mental Disorders, Fifth Edition (DSM-5) [12], may result in measurement variance between countries. Expressions of distress vary significantly between cultures, influencing the phenomenology of mental illness resulting in differences in illness presentations. Research on the validity of DSM-5 diagnoses in adolescents living in the Global South is limited [13]. Categorical diagnoses have limitations when measuring population mental health. Mental illness is dimensional and diagnostic thresholds are somewhat arbitrary. However, diagnoses have the advantage of identifying those individuals whose mental health symptoms are both persistent and impairing.

Whilst problems in diagnostic validity across cultures may result in an underestimate of adolescent mental disorder prevalence in LMICs, it is less likely that behaviourally-orientated items about self-harm or suicide or questions about ACEs would be misinterpreted. There were very low rates of endorsement of these items in Indonesia and Vietnam as compared to Kenya, suggesting a true prevalence difference between countries, noting that ACEs are risk factors for mental disorders. The between country variation in the prevalence of risk factors, suicidal behaviours, and mental disorders are remarkably consistent and match the relatively low rates of suicidal thoughts previously reported in adolescents from Asian countries [14].

Mental illness is multi-dimensional with many adolescents experiencing sub-diagnostic and transdiagnostic symptoms, which cause significant distress and impairment. The threshold required for the diagnosis of a mental disorder according to DSM-5 may result in under-recognition of a significant proportion of the population who are symptomatic. Responses to individual items on the DISC-5 from NAMHS are available and will be analysed in future studies.

The low prevalence of adolescent mental disorders in Indonesia and Vietnam suggests there is much that the Global North can learn from the Global South. Substantial investment in mental health services in high-resource settings have failed to improve population mental health [15]. A 2011 review reported the effective implementation into primary health care services of mental health care for children living in low resource settings [16]. Improving population mental health requires a public health approach with promotion of strategies to promote wellbeing, prevention of exposure to risk factors particularly in early years of life, and ensuring people living with mental disorders are supported and experience a sense of purpose [17]. Further research is needed to understand factors which are responsible for the inter-country variation in prevalence and identify risk and protective factors which can inform population-based preventative interventions.

## Stigma

Stigma, which encompasses the experience of negative attitudes and beliefs resulting from mental illness, may be internalized (self-stigma) or propagated by others including family members, peers, health care workers and the wider community [18]. Stigma is a significant impediment to the measurement of mental disorders in in LMICs, as individuals fear revelations about their illness may bring shame upon themselves or their families or they may be judged negatively by others.

Whilst it is possible that stigma influenced participant responses in NAMHS, strategies were implemented to minimize this risk. Experienced interviewers were comprehensively trained, privacy of all participants ensured (including for the adolescent from the primary caregiver), and substantial community consultation was undertaken in sampled areas prior to the commencement of data collection. Questions on particularly sensitive issues were self-administered by the adolescent.

## Importance of measurement

NAMHS provides population-based data on mental disorders that are essential to supporting the 2030 Sustainable Development Agenda. Target 3.4 aims to reduce premature mortality from non-communicable diseases by one third through promotion, prevention, and treatment targeting mental health and well-being [19]. Measurement of adolescent mental disorders has been identified as a priority in improving global adolescent health [20]. Two other initiatives are underway to provide population-based data on mental health in adolescents living in low-resource settings. The United Nations Children’s Fund (UNICEF) has implemented the Measuring Mental Health Among Adolescents at the Population Level (MMAPP) project (20), which aims to use symptom scales to estimate the prevalence of mental health problems in low-resource settings. Additionally, the World Health Organization (WHO) is undertaking the Global Action for Measurement of Adolescent Health (GAMA) Initiative, which aims to identify and define a core set of adolescent health indicators including those related to mental health [21].

## Conclusion

NAMHS significantly advances knowledge of adolescent mental health in the Global South. High quality, large-scale epidemiological research is critical to informing health policy. This must be complemented by the development and validation of measures that capture the wide-ranging symptoms of distress experienced in different cultures and health service research to examine the effectiveness and cost effectiveness of interventions in low resource settings [22]. Repeated population-based surveys from over a decade ago revealed a striking increase in the prevalence of mental disorders in female adolescents living in high-income countries [23]. NAMHS will enable trends of mental health in the Global South to be tracked in the years to come. This will be increasingly important as the world faces ongoing challenges such as climate change [24] and unforeseen events such as the COVID-19 pandemic [25] which will inevitably affect global mental health.

## Data Availability

The NAMHS datasets are co-owned by the University of Queensland (UQ) and each respective in-country lead organisation (K-NAMHS: African Population and Health Research Center [APHRC] and UQ; I-NAMHS: UGM and UQ; V-NAMHS: Institute of Sociology [IOS] and UQ). Currently, these datasets or analysis of these datasets are available for collaborative work on request to the relevant data owners following an established protocol. Work is currently underway to convert the NAMHS datasets into public use datasets, allowing for wide use of these datasets while ensuring protection of participant privacy, adherence to country-specific legislation, and appropriate use of data. This includes development of accompanying meta-data, inclusive of a codebook, technical manual, and analysis files. The expected launch date for these public use datasets and accompanying meta-data is 2026, with hosting mechanisms currently under development in line with country legislation and ethical requirements.

## Abbreviations

ACEs: Adverse childhood experiences
DISC-5: Diagnostic Interview Schedule for Children, Version 5
DSM-5: Diagnostic and Statistical Manual of Mental Disorders, Fifth Edition
GAMA: Global Action for Measurement of Adolescent Health
LMICs: Low- and middle-income countries
MMAPP: Measuring Mental Health Among Adolescents at the Population Level
NAMHS: National Adolescent Mental Health Surveys
UNICEF: United Nations Children’s Fund
WHO: World Health Organization

## References

[1] GBD 2019 Mental Disorders Collaborators. Global, regional, and National burden of 12 mental disorders in 204 countries and territories, 1990–2019: a systematic analysis for the global burden of disease study 2019. Lancet Psychiatry. 2022;9(2):137–50. 35026139 10.1016/S2215-0366(21)00395-3 PMC8776563

[2] Solmi M, Radua J, Olivola M, Croce E, Soardo L, Salazar de Pablo G, et al. Age at onset of mental disorders worldwide: large-scale meta-analysis of 192 epidemiological studies. Mol Psychiatry. 2022;27(1):281–95. 34079068 10.1038/s41380-021-01161-7 PMC8960395

[3] Erskine HE, Baxter AJ, Patton G, Moffitt TE, Patel V, Whiteford HA, et al. The global coverage of prevalence data for mental disorders in children and adolescents. Epidemiol Psychiatr Sci. 2017;26(4):395–402. 26786507 10.1017/S2045796015001158 PMC6998634

[4] United Nations Department of Economic and Social Affairs. Population division. World population prospects 2022. United Nations; 2022.

[5] Erskine HE, Maravilla JC, Wado YD, Wahdi AE, Loi VM, Fine SL, et al. Prevalence of adolescent mental disorders in Kenya, Indonesia, and Viet Nam measured by the National adolescent mental health surveys (NAMHS): a multi-national cross-sectional study. Lancet. 2024;403(10437):1671–80. 38588689 10.1016/S0140-6736(23)02641-7

[6] Wado YD, Njeri A, Odunga SA, Akuku I, Wahdi AE, Fine SL et al. The association between adverse childhood experiences and mental disorders among adolescents in Kenya, Indonesia, and Vietnam: Evidence from the National Adolescent Mental Health Surveys. Child Adolesc Psychiatry Ment Health. 2025;19(1). 10.1186/s13034-025-00919-z PMC12312255 40739645

[7] Erskine HE, Maravilla J, Fine SL, Ramaiya A, Li M, Wahdi AE et al. Bullying victimisation and perpetration and the association with mental disorders among adolescents in Kenya, Indonesia, and Vietnam: Findings from the National Adolescent Mental Health Surveys. Child Adolesc Psychiatry Ment Health. 2025;19(1). 10.1186/s13034-025-00922-4 PMC12312254 40739252

[8] Fine SL, Ramaiya A, Li M, Wahdi AE, Wilopo SA, Loi VM et al. Mental health among sexually and gender diverse adolescents in Indonesia and Vietnam: Results from the National Adolescent Mental Health Surveys. Child Adolesc Psychiatry Ment Health. 2025;19(1). 10.1186/s13034-025-00921-5 PMC12312252 40739519

[9] Wahdi AE, Putri YA, Setyawan A, Fine SL, Ramaiya A, Li M et al. Mental health service use among adolescents in three low- and middle-income countries: An analysis of the National Adolescent Mental Health Surveys. Child Adolesc Psychiatry Ment Health. 2025;19(1). 10.1186/s13034-025-00924-2 PMC12312253 40739641

[10] Maravilla JC, Fine SL, Ramaiya A, Li M, Wado YD, Wahdi AE et al. Social support and mental health among adolescents in Kenya, Indonesia, and Vietnam: A latent class analysis using the National Adolescent Mental Health Surveys. Child Adolesc Psychiatry Ment Health. 2025;19(1). 10.1186/s13034-025-00923-3 PMC12312257 40739525

[11] Erskine HE, Blondell SJ, Enright ME, Shadid J, Wado YD, Wekesah FM, et al. Measuring the prevalence of mental disorders in adolescents in Kenya, Indonesia, and Vietnam: study protocol for the National adolescent mental health surveys. J Adolesc Health. 2023;72(1s):S71–8. 36229399 10.1016/j.jadohealth.2021.05.012

[12] American Psychiatric Association. Diagnostic and Statistical Manual of Mental Disorders, Fifth Edition. Arlington, VA: American Psychiatric Association; 2013.

[13] Canino G, Alegría M. Psychiatric diagnosis - is it universal or relative to culture? J Child Psychol Psychiatry. 2008;49(3):237–50. 18333929 10.1111/j.1469-7610.2007.01854.x PMC3104469

[14] Evans E, Hawton K, Rodham K, Deeks J. The prevalence of suicidal phenomena in adolescents: a systematic review of population-based studies. Suicide Life Threat Behav. 2005;35(3):239–50. 16156486 10.1521/suli.2005.35.3.239

[15] Jorm AF. Australia’s ‘Better Access’ scheme: has it had an impact on population mental health? Aust N Z J Psychiatry. 2018;52(11):1057–62. 30284914 10.1177/0004867418804066

[16] Skokauskas N, Belfer M. Global child mental health: what can we learn from countries with limited financial resources? Int Psychiatry. 2011;8(2):45–7. 31508080 PMC6735009

[17] Patel V, Saxena S, Lund C, Kohrt B, Kieling C, Sunkel C, et al. Transforming mental health systems globally: principles and policy recommendations. Lancet. 2023;402(10402):656–66. 37597892 10.1016/S0140-6736(23)00918-2

[18] Mascayano F, Armijo JE, Yang LH. Addressing stigma relating to mental illness in Low- and Middle-Income countries. Front Psychiatry. 2015;6:38. 10.3389/fpsyt.2015.00038. 10.3389/fpsyt.2015.00038 PMC4355984 25814959

[19] World Health Organization. Guidelines on mental health promotive and preventive interventions for adolescents: helping adolescents thrive. Geneva: World Health Organization; 2020. Licence: CC BY-NC-SA 3.0 IGO. 33301276

[20] Carvajal-Velez L, Harris Requejo J, Ahs JW, Idele P, Adewuya A, Cappa C, et al. Increasing data and Understanding of adolescent mental health worldwide: UNICEF’s measurement of mental health among adolescents at the population level initiative. J Adolesc Health. 2023;72(1s):S12–4. 36229402 10.1016/j.jadohealth.2021.03.019

[21] Guthold R, Moller AB, Azzopardi P, Ba MG, Fagan L, Baltag V, et al. The global action for measurement of adolescent health (GAMA) Initiative-Rethinking adolescent metrics. J Adolesc Health. 2019;64(6):697–9. 31122505 10.1016/j.jadohealth.2019.03.008 PMC6531407

[22] Skokauskas N, Fung D, Flaherty LT, Klitzing K, Puras D, Servili C, et al. Shaping the future of child and adolescent psychiatry. Child Adolesc Psychiatry Ment Health. 2019;13:19. 31007713 10.1186/s13034-019-0279-y PMC6458731

[23] Bor W, Dean AJ, Najman J, Hayatbakhsh R. Are child and adolescent mental health problems increasing in the 21st century? A systematic review. Aust N Z J Psychiatry. 2014;48(7):606–16. 24829198 10.1177/0004867414533834

[24] Crandon TJ, Scott JG, Charlson FJ, Thomas HJ. A social–ecological perspective on climate anxiety in children and adolescents. Nat Clim Change. 2022;12(2):123–31.

[25] COVID-19 Mental Disorders Collaborators. Global prevalence and burden of depressive and anxiety disorders in 204 countries and territories in 2020 due to the COVID-19 pandemic. Lancet. 2021;398(10312):1700–12. 34634250 10.1016/S0140-6736(21)02143-7 PMC8500697

